# A Height-Based Dosing Algorithm of Bupivacaine in Spinal Anesthesia for Decreasing Maternal Hypotension in Cesarean Section Without Prophylactic Fluid Preloading and Vasopressors: A Randomized-Controlled Non-Inferiority Trial

**DOI:** 10.3389/fmed.2022.858115

**Published:** 2022-06-10

**Authors:** Qiang Huang, Gengzhi Wen, Chao Hai, Zihao Zheng, Yali Li, Zengping Huang, Bowan Huang

**Affiliations:** ^1^Department of Anesthesiology, ShenZhen People's Hospital, Second Clinical Medical College of Jinan University, Shenzhen, China; ^2^Department of Anesthesiology, Shenzhen Traditional Chinese Medicine Hospital, The Fourth Clinical Medical College of Guangzhou University of Chinese Medicine, Shenzhen, China

**Keywords:** anesthesia, spinal, bupivacaine, cesarean section, height, hypotension

## Abstract

**Background:**

There is a high incidence of maternal hypotension in spinal anesthesia for cesarean section. The aim of the study is to investigate whether there is a height-based dosing algorithm of bupivacaine that provides adequate anesthesia with less maternal hypotension.

**Methods:**

There were 2 groups of 280 parturients who did not receive prophylactic fluid preloading: Test and Conventional group. In Test group, a height based dosing algorithm was used to confirm the dose of bupivacaine in parturients without prophylactic vasopressors. In the Conventional group, a constant dose of bupivacaine was used. The complications and quality of anesthesia were evaluated.

**Results:**

In the Conventional group, the shorter participants had higher incidence of hypotension, faster sensory block time, and more participants with complete motor block (*p* = 0.030, 2.957 × 10^−14^, and 0.012). In the Test group, the incidence of hypotension, sensory block time, and number of participants with complete motor block did not change with height (*p* = 0.199, 0.617, and 0.209). The height-based dosing algorithm of bupivacaine decreased the incidence of hypotension (*p* = 0.004), induced lower sensory block level and less degree of motor block (*p* = 3.513 × 10^−7^ and 5.711 × 10^−11^). The quality of analgesia, quality of muscle relaxation, and degree of intraoperative comfort were similar in both groups (*p* = 0.065, 0.498, and 0.483).

**Conclusions:**

The height influences the dose of bupivacaine in spinal anesthesia; without prophylactic fluid pre-loading and vasopressors, the height-based dosing algorithm of bupivacaine is suitable, and meets the cesarean section' requirement with less maternal hypotension.

**Clinical Trial Registration:**

www.ClinicalTrials.gov, identifier: NCT03497364.

## Introduction

Spinal anesthesia is popularly applied for cesarean section due to high-quality anesthesia and no inhibitory effect of general anesthetics on the fetus (1, 2). Unfortunately, there is a high incidence of maternal hypotension, which is attributed to special physiological changes in parturients (3) and sympathetic block (1). Mild hypotension may result in a series of side effects [e.g., hypoxemia and acidosis in fetus (4), and nausea, vomiting, and dizziness in parturient] (5). For severe hypotension, the life of the parturient and fetus may be threatened (6). In obstetric anesthesia, it has been deemed to be the Holy Grail for effectively preventing or treating maternal hypotension resulted from spinal anesthesia (2).

For decreasing the maternal hypotension, the fluid preloading (colloid or crystalloid) (7) and/or vasopressors (ephedrine or phenylephrine) (8) is often prophylactically used. In late pregnancy, the blood volume and cardiac load of the parturient significantly increase, which may be further exacerbated by fluid preloading. Ephedrine may increase the incidence of fetal acidosis (9), which may be associated with poor neonatal outcome (10). Phenylephrine may induce bradycardia (11), and decrease cardiac output (8). Consequently, for parturient or fetus, it may be beneficial that avoiding prophylactic fluid preloading and/or vasopressors.

It is controversial whether the patient height is related to the block level for spinal anesthesia. In several studies, there is no statistical association between block level and height (12, 13). The dose of the local anesthetic does not change with height in many studies (1, 4, 14). However, vertebral column length can influence the block level (15). In Norris's study, the height accounts for 10.6% of the variation in the length of the spine, there is a statistical correlation between vertebral column length and height (13). Thus, the block level theoretically depends on height, which is verified in two studies (16, 17). In spinal anesthesia, as the dose of local anesthetic decreases, the block level lowers, the maternal hypotension decreases, but inadequate muscle relaxation and analgesia may increase (18). Based on above analysis, we hypothesize that in spinal anesthesia, even without prophylactic fluid preloading and vasopressors, there is a height-based dosing algorithm of local anesthetic that provides adequate anesthesia for cesarean section with less maternal hypotension.

To test our hypothesis, for cesarean section, spinal anesthesia with bupivacaine was carried out, the dose of bupivacaine was adjusted according to height in this study. In this manner, for cesarean section, we attempted to found a suitable dose of bupivacaine in spinal anesthesia.

## Materials and Methods

### General

**Figure 1:**
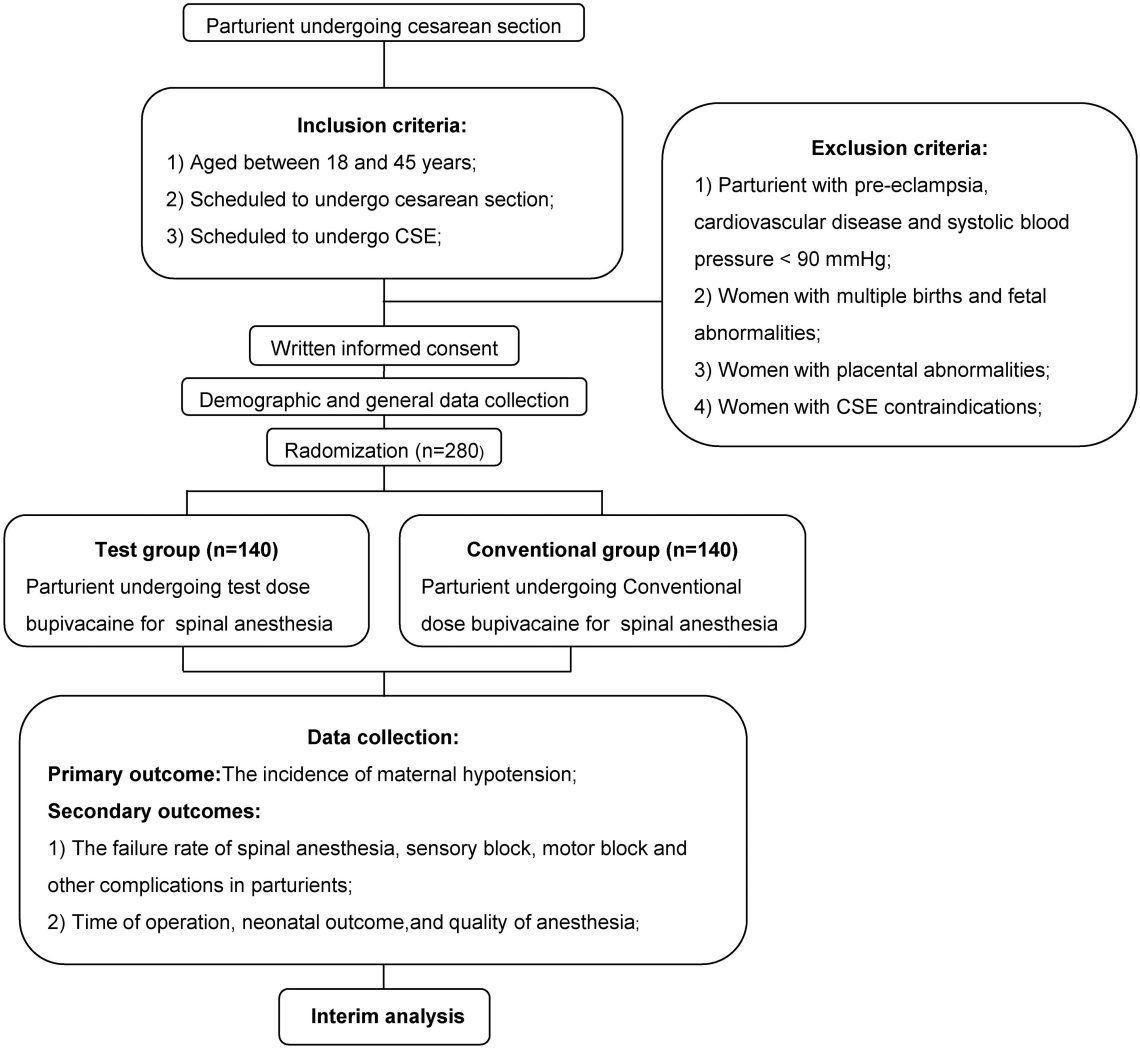
Study flow chart. CSE, combined spinal–epidural.

Ethical approval for this study protocol was obtained from the Ethics Committee of Shenzhen People's Hospital of Jinan University, and this study was registered at ClinicalTrials.gov on April 12, 2018 (NCT03497364). The full protocol was available in pubmed (https://www.ncbi.nlm.nih.gov/pubmed/31101694). Figure 1 provided the study flow chart. Parturients (scheduled for cesarean section, aged 18–45 years) were recruited after February 8, 2018, and were randomly divided into 2 groups, Test and Conventional groups. Before anesthesia, all parturients were prohibited to drink clear liquids for 2–3 h, and eat non-fatty solids for 6–8 h. Written informed consent was acquired from all parturients. Parturients with pre-eclampsia, cardiovascular disease, systolic blood pressure (SBP) < 90 mmHg, multiple births, placental abnormalities, fetal abnormalities, and combined spinal-epidural anesthesia (CSE) contraindications were excluded from the study.

### Intervention

Before entering the operation room (OR), the heart rate (HR), and blood pressure of the parturients were measured. Once entering the OR, electrocardiogram, HR, blood pressure, and SPO_2_ were monitored. Supplementary oxygen (2 L/min) was given via a facemask. In the forearm vein, venipuncture was carried out. Then, Ringer's lactate (1,000 ml) was slowly infused into parturients in both groups (2 ml/kg/h).

To furthest decrease incomplete analgesia and muscle relaxation, we performed CSE instead of spinal anesthesia in this study. CSE was performed at the L3–4 interspace in left lateral position by the experienced doctors, who had been trained about how to more identically perform CSE before starting this study. Isobaric bupivacaine was marketed in our hospital. We were accustomed to use isobaric bupivacaine in spinal anesthesia for cesarean section all the time. In the Test group, 1.15–1.7 ml isobaric bupivacaine (5 mg/ml) from ChaoHui drug company (ShangHai, China) was applied. The bupivacaine dose was adjusted according to the height of the parturients (0.05 ml/2–3 cm, Table 1) (19). In the Conventional group, 1.8 ml isobaric bupivacaine (5 mg/ml) was applied (20). The direction of side opening on spinal needle was toward the cephalic in both groups. After intrathecal injection, the parturients were immediately placed the supine position with a left lateral tilt (15 degree). Ringer's lactate was quickly infused in both groups (10 ml/kg/h) (21). Prophylactic phenylephrine was infused via micropump (0.25 μg.kg^−1^.min^−1^ (i.e., 2.5 ml/h) in the Conventional group (22). Normal saline was infused (2.5 ml/h) in the Test group. Prophylactic fluid preloading was not applied for all participants.

**Table 1:** The relationship between the height of the parturient and dose of 0.5% bupivacaine.

**Height of parturient (cm)**	**Dose of 0.5% bupivacaine (ml)**
173–174	1.70
170–172	1.65
168–169	1.60
165–167	1.55
163–164	1.50
160–162	1.45
158–159	1.40
155–157	1.35
153–154	1.30
150–152	1.25
148–149	1.20
145–147	1.15

*This table was from the published full protocol in BMJ Open (https://www.ncbi.nlm.nih.gov/pubmed/31101694)*.

Maternal hypotension was defined by SBP <90 mmHg or 70% of baseline value. From anesthesia initiation to delivery, when maternal hypotension occurred, this parturient was defined as a parturient with hypotension. Maternal hypotension was treated with phenylephrine (100 μg). Bradycardia (<60 beat/min) was treated with atropine (0.5 mg). Nausea and vomiting were treated with metoclopramide (10 mg). According to anatomical structure, for cesarean section, it is recommended that the highest sensory block level should reach dermatome level dominated by the fourth thoracic nerve (T4) (23). However, in different studies, the highest sensory block level is required to reach T4, T5, T6, or T8 (5, 14, 20, 24–26). In some parturients, even though the highest sensory block level reaches T4, they still feel slight pain (23). In our clinical practice, when the highest sensory block level reaches T8 at 10 min after anesthesia, the anesthesia is adequate. Therefore, at 10 min after anesthesia, if the sensory block level did not reach T8, spinal anesthesia was regarded as a failure (24, 25). The parturients without successful spinal anesthesia were excluded from the study. For parturients without successful spinal anesthesia, 2% lidocaine + 0.75% ropivacaine (15 ml) was given via epidural space until the level of sensory block is not lower than T8 (24, 25) or the anesthetic technique was changed to general anesthesia. For parturients with successful spinal anesthesia, when the parturients felt pain after taking out the fetus, fentanyl (0.1 mg) via a vein and/or 2% lidocaine + 0.75% ropivacaine (15 ml) via the epidural space were carried out.

### Data Acquisition

Before anesthesia, demographic data, baseline data, and general data were recorded. After bupivacaine injection, the HR, blood pressure, respiratory rate and SPO_2_ were immediately collected. The level of sensory block was measured via hypoalgesia. If the hypoalgesia level reached T8, anesthesia was considered to be sufficient for cesarean section (24, 25). Motor block was evaluated with the modified Bromage scale (26).

After taking out the fetus, APGAR scores at 1 and 5 min were assessed. For blood gas analysis, blood sample was taken from umbilical artery. The complications (hypotension, dizziness, nausea, vomiting, dyspnea, and bradycardia) were recorded. After cesarean section, the time from anesthesia initiation to skin incision, time from skin incision to delivery and operation duration were computed. The quality of analgesia (judged by the anesthetist), the quality of muscle relaxation (judged by the surgeon) and the degree of intraoperative comfort (judged by the patient via asking how you feel during operation) were recorded as excellent, good, fair, or poor (14).

### Statistical Analysis

#### Sample Size Calculation

For maternal hypotension, there is an incidence of 30% in Geng et al.'s study (27). A ≥ 15% difference in the incidence of maternal hypotension was considered to be significant in a clinical setting. A non-inferiority one-sided test was performed with this equation ($n=\frac{2\;•\;p\;•\;\left(1\;-\;p\right)\;•\;{\left(z_{\left(1-\alpha \right)}\;+\;z_{\left(1-\beta \right)}\right)}_{}^{2}}{\Delta_{}^{2}}$) for sample size calculation (28). Assuming a power of 0.80 and a type I error protection of 0.05, 116 subjects were required in each group. To compensate for dropouts and protocol violations, we planned to recruit at least 280 parturients in this study.

#### Outcome Analysis

Statistical analysis was performed using SPSS 13.0 software package. All continuous data were presented as the mean (*SD*). With chi-square test, the enumeration data were analyzed. With Student's *t*-test (Normally distributed data) or Mann-Whitney U-test/Kruskai-Wallis H (Non-normally distributed data), the continuous data were analyzed. A *p*-value <0.05 were deemed significant.

## Results

### Characteristics of Parturients

This study excluded 13 parturients from the Test group and 9 parturients from the Conventional group due to a variety of factors (e.g., unsuccessful spinal anesthesia and protocol violations). The demographic data, general data, baseline data, and concomitant disease of parturients were similar in both groups (Table 2).

**Table 2:** Parturient characteristics.

	**Test group (*n* = 127)**	**Conventional group (*n* = 131)**	***Z*, *t* or *χ^2^***	***p***
Age (years)	32.181 (5.417)	31.122 (4.785)	−1.665	0.096
Height (cm)	158.854 (4.875)	158.657 (4.834)	0.327^#^	0.744
Weight (kg)	67.053 (9.551)	68.221 (10.249)	−0.395	0.693
Weeks of gestation	37.504 (2.407)	37.939 (2.063)	−1.854	0.064
Previous cesarean	71	67	0.412	0.521
Initial SBP (mmHg)	123.221 (16.468)	119.412 (14.533)	−1.509	0.131
Initial HR (beats/min)	86.969 (13.751)	84.336 (13.138)	−1.704	0.084
Time from anesthesia initiation to skin incision (min)	19.024 (7.411)	19.046 (7.651)	−0.177	0.860
Time from skin incision to delivery (min)	8.394 (4.794)	7.565 (4.127)	−1.711	0.087
Operation duration (min)	55.732 (15.938)	54.710 (13.967)	−0.153	0.878
**Concomitant disease**				
Hypertension	10	9	0.005	0.944
Diabetes	25	23	0.078	0.780
HGB <90 g/L	3	7	0.842	0.359
Hyperthyroidism	2	1	0.001	0.978
Hypothyroidism	1	4	0.754	0.385
Abnormal liver function	1	0	2.414 × 10^−4^	0.988
Macrosomia	0	2	0.473	0.491

# *Indicated that Student's t-test was used. For other continuous data, Mann-Whitney U-test was used*.

### Complications, Sensory Block, and Motor Block of Parturients

The incidence of hypotension (primary outcome), dizziness, nausea, dyspnea and bradycardia, and number of hypotensive recordings were fewer in Test group than those in Conventional groups (Table 3). The incidence of vomiting was no statistically different in both groups (Table 3). The sensory block levels of three parturients in Test group and two parturients in Conventional groups were lower than T8 at 10 min after anesthesia (Table 4). For sensory block, in comparison with both in Test groups, the time for sensory block to reach T8 (Time _sensoryblocktoT8_) was faster, and the sensory level at 10 min after anesthesia was higher in the Conventional group (Table 4). For motor block, 15 parturients in Test group and two parturients in the Conventional groups could not reach complete block, and not be included to compute the time to complete motor block (Time _completemotorblock_). In comparison with both in Test groups, the Time _completemotorblock_ was faster, and the numbers of parturients with complete motor block at 10 min after anesthesia (Number _completemotorblock_) were more in Conventional group (Table 4).

**Table 3:** Incidence of side effects in parturients.

	**Test group (*n* = 127)**	**Conventional group (*n* = 131)**	***χ^2^***	***p***
Hypotension	18	39	8.231	0.004
**Number of hypotensive recordings**				
0	109	92	14.268	0.003
1–2	16	22		
3–4	2	15		
≥5	0	2		
Dizziness	6	17	4.440	0.035
Nausea	3	12	4.272	0.039
Vomiting	1	6	2.224	0.136
Bradycardia	4	15	5.353	0.021
Dyspnea	0	7	5.098	0.024

**Table 4:** Characteristics of spinal anesthesia.

	**Test group (*n* = 127)**	**Conventional group (*n* = 131)**	***Z* or *χ^2^***	***p***
Unsuccessful spinal anesthesia	3	2	0.001	0.979
Time _sensoryblocktoT8_ (min)	4.858 (1.521)	3.733 (1.583)	−6.029	1.647 × 10^−9^
**Sensory level at 10 min after anesthesia**				
>T2	2	16	42.883	3.513 × 10^−7^
T2	9	25		
T3	13	29		
T4	24	24		
T5	36	20		
T6	26	14		
T7	13	3		
T8	4	0		
Time _completemotorblock_ (min)	13.053 (7.115)	6.674 (5.400)	8.948	3.618 × 10^−19^
Number _completemotorblock_	61	113	41.194	1.379 × 10^−10^

In Test group, the incidence of hypotension, Time _sensoryblocktoT8_ and Number _completemotorblock_ were similar in parturients with different height (Table 5). In Conventional group, as the height increased, the incidence of hypotension and Number _completemotorblock_ decreased, the Time _sensoryblocktoT8_ increased (Table 5).

**Table 5:** Comparison among different heights in Test and Conventional groups.

		**Test group**				**Conventional group**		
	***n***	**Hypotension**	**Time _**sensoryblocktoT8**_ (min)**	**Number _**completemotorblock**_**	***n***	**Hypotension**	**Time _**sensoryblocktoT8**_ (min)**	**Number _**completemotorblock**_**
145–149 cm	1	1	/	0	3	3	1.333 (0.577)	3
150–154 cm	22	2	5.136 (1.781)	13	20	8	2.400 (0.503)	18
155–159 cm	45	6	4.956 (1.445)	17	49	17	3.286 (0.816)	42
160–164 cm	42	6	4.690 (1.423)	24	46	9	4.239 (1.079)	38
165–169 cm	15	3	4.667 (1.759)	7	12	2	6.500 (1.624)	6
170–174 cm	2	0	4.000 (0.000)	0	1	0	/	0
*χ^2^*		7.298	2.657	7.154		12.347	69.456	14.664
*P*		0.199	0.617	0.209		0.030	2.957 × 10^−14^	0.012

*/ indicated that the mean could not be obtained, because the sample size was 1*.

### Quality of Anesthesia and Neonatal Outcome

For quality of analgesia, although “good” parturients were more in Test group, there was no statistical difference between 2 groups (Table 6). For “good” parturients, no matter whether the highest sensory block level reaches T4, they usually felt slight transitory pain during taking out the fetus. This slight transitory pain was related with pressing the uterus by surgical assistant, and could be completely endured by the parturients. The quality of muscle relaxation and degree of intraoperative comfort were similar in both groups (Table 6). As for neonatal outcome, there was no statistical difference in both groups (Table 7).

**Table 6:** Quality of anesthesia.

	**Quality of analgesia**		**Quality of muscle relaxation**		**Degree of intraoperative comfort**	
	**Test group** **(*n* = 127)**	**Conventional group** **(*n* = 131)**	**Test group** **(*n* = 127)**	**Conventional group** **(*n* = 131)**	**Test group** **(*n* = 127)**	**Conventional group** **(*n* = 131)**
Excellent	106	122	114	124	104	101
Good	17	7	7	4	16	25
Fair	2	2	3	2	5	3
Poor	2	0	3	1	2	2
*χ^2^*	7.229		2.377		2.458	
*P*	0.065		0.498		0.483	

**Table 7:** Neonatal outcome.

	**Test group (*n* = 127)**	**Conventional group (*n* = 131)**	***Z* or *χ^2^***	***p***
Male	72	70	0.161	0.689
Weight (kg)	3.120 (0.552)	3.136(0.513)	−0.338	0.735
1 min Apgar score	9.882 (0.544)	9.863 (0.642)	−0.276	0.783
5 min Apgar score	9.976 (0.198)	9.977 (0.195)	−0.031	0.975
**Blood gas analysis**				
PH	7.276(0.043)	7.229 (0.442)	−0.654	0.513
PO_2_	17.535 (4.203)	17.977 (4.398)	−0.512	0.608
PCO_2_	52.535 (6.185)	54.557 (8.667)	−1.928	0.054
BE	−2.244 (2.298)	−2.137 (2.411)	−0.486	0.627

## Discussion

### Potential Factors Influenced Dose of Bupivacaine

In comparison with patients in other surgical department (e.g., orthopedics department), a relatively small dose of bupivacaine can induce a higher sensory block level in spinal anesthesia for cesarean section (20). That is, the parturient is more sensitive to the dose of bupivacaine, which should be adjusted based on some factors. Weight is a controversial factor. There are some studies showed that the dose of bupivacaine should (29, 30) or not (13, 31, 32) be adjusted according to weight. In addition, in some studies, only in parturients with high body mass index, weight is an interference factor (33, 34). In our practice, parturients with high body mass index are small, and weight does not seem to influence the block level. Theoretically, the injection speed can influence the spread of bupivacaine, but this is not found in clinical practice (35). Age is also an interference factor, but the interference effect occurs only in the elderly (36, 37). The parturients are young. The direction of side opening on spinal needle, position of parturients and punctured interspace may also influence the spread of bupivacaine (15, 38). However, we had identically limit that the direction of side opening was toward the cephalic, the parturients were immediately placed the supine position with a left lateral tilt after intrathecal injection and the punctured interspace was L3–4. Increasing evidences show that height is an important factor influenced the dose of bupivacaine (16, 17, 30, 39). Furthermore, height is a continuous variable, has a large change range in parturients. Therefore, height was selected as the only adjusted factor for the dose of bupivacaine in this study.

### Dose of Bupivacaine Depended on Height

The height is related with vertebral column length (13). The vertebral column length can influence the block level (13), which is associated with the injected dose of local anesthetic in the subarachnoid space (18). In addition, in parturients, high abdominal pressure decreases the volume of the subarachnoid space (40, 41). If the dose of bupivacaine is constant, the incidence of hypotension, Time _sensoryblocktoT8_ and Number _completemotorblock_ changed with height (Table 5). Therefore, the height influences the block level, which is consistent with the results in 2 studies (16, 17). That is, the dose of bupivacaine depends on height, and should be adjusted according to height. When the dose of bupivacaine changed with height, the incidence of hypotension, Time _sensoryblocktoT8_ and Number _completemotorblock_ changed little in parturients with different height (Table 5). Therefore, the height based dosing algorithm of bupivacaine in this study is reasonable, especially using a low dose of bupivacaine (16, 17).

The height of parturient is associated with the block onset time (42) and block level (29), and is regarded as a risk factor for hypotension (29). However, in some studies, the variation in block spread of the subjects with same height is very large, the height does not influence the block level of spinal anesthesia (13, 31). This may be due to that the dose of bupivacaine is more in these studies than it in our study, and the effect of height on block level is undetectable (39).

### Height Based Dosing Algorithm of Bupivacaine Induced a Low Incidence of Complications Even Without Prophylactic Fluid Pre-loading and Vasopressors

In spinal anesthesia, the motor and sensory block levels depend on the dose of local anesthetic (18, 27). In comparison with the Conventional group, the dose of bupivacaine was adjusted according to height and was smaller in Test group. Therefore, the Time _sensoryblocktoT8_ or Time _completemotorblock_ increased the sensory block level at 10 min or Number _completemotorblock_ decreased in Test group (Table 4). This implies that the degree of sympathetic block was deeper, and the range of sympathetic block was wider in the Conventional group than both in Test group. The hypotension depends on the range and degree of a sympathetic block (1). Therefore, the maternal hypotension was less in Test group (Table 3).

Other complications are often correlated with hypotension (5). Thus, the incidence of other complications also decreased in Test group (Table 3), which is consistent with previous studies (5). Theoretically, hypotension may decrease blood flow volume of umbilical artery, and induce hyoxemia and acidosis in fetus (4). Although the incidence of hypotension was higher in Conventional group (Table 3), the hypotension were timely rectified with phenylephrine. The neonatal outcome was not different in both groups (Table 7) (19).

### Height Based Dosing Algorithm of Bupivacaine Provided Adequate Anesthesia

Theoretically, the highest sensory block level should reach T4 for adequate analgesia in cesarean section (5, 23). Actually, the requirement of highest sensory block level is T4, T5, T6, or T8 in different studies (5, 14, 20, 24–26). In this study, the highest sensory block level was required to be T8. In previous experience of other researchers (23) and in this study, even though the highest sensory block level reaches T4, some parturients still feel slight pain. The incidence of pain in this study (Table 6) was similar to it in other studies (34). No matter whether the highest sensory block level reaches T4, the pain usually occurred during taking out the fetus. We consider this pain was mostly attributed to pressing the uterus by surgical assistant, and was not related with the sensory block level. Our results showed that T8 was suitable requirement of highest sensory block level. This may be partly due to 2 reasons. Firstly, before taking out the fetus, the operative region locates on anesthesia of abdomen and, and is relatively narrow. Secondly, after taking out the fetus, we timely applied analgesic via a vein or local anesthetic via the epidural space.

For quality of analgesia, although more “good” parturients felt slight transitory pain in Test group, there was no statistical significance between 2 groups (Table 6) and these parturients could completely endure this pain. Moreover, the sensory block level ≥ T8 is taken as adequate analgesia for cesarean section (24, 25). In Test groups, the sensory block level could reach T8 at 10 min after anesthesia in most parturients (Table 4). Consequently, we consider the height based dosing algorithm of bupivacaine provides adequate analgesia.

In Test group, although the Number _completemotorblock_ is less, the motor block level could reach modified Bromage scale = 2 in all parturients. The quality of muscle relaxation in Test group was similar to it in Conventional group (Table 6). In addition to pain, the degree of intraoperative comfort is also related with other complications (e.g., nausea, vomiting, dizziness, and dyspnea). Although parturients with slight pain were more in Test group (Table 6), parturients with other complications were more in Conventional group (Table 3). The degree of intraoperative comfort was similar in both groups (Table 6). Taken together, the height based dosing algorithm of bupivacaine provides adequate anesthesia, which is further supported by that smaller dose of bupivacaine in spinal anesthesia can meet the requirement of cesarean section (20).

In comparison with it in Conventional group, the dose of bupivacaine in Test group was less, and the time to reach adequate anesthesia was later (Table 4). This is supported by other studies (30, 42). However, the height based dosing algorithm of bupivacaine did not delay the operation duration, because the time to reach adequate anesthesia was 4.858 (1.521) min, which was approximate to the time of skin disinfection, placing sterile surgical drape and wearing sterile surgical clothes for surgeon. Furthermore, the parturients included in this study were not in extreme critical situation. In Harten et al.' study (29), for parturients in extreme critical situation emergency, the height-based dosing algorithm of bupivacaine is not recommended, because the time to reach adequate anesthesia is longer than it in our study. This time difference may be partly attributed to the different definition of adequate anesthesia and racial difference (29).

### Strengths and Limitations

In spinal anesthesia, we clarified the relation between the parturient height and bupivacaine dose, and verified it is feasible that spinal anesthesia for cesarean section is carried out under condition of no prophylactic fluid pre-loading and vasopressors. Our study helps to decrease the dangerousness of parturients and fetuses with lower incidence of complications, and alleviate the stress of anesthetist.

There are two limitations in this study. First, the number of parturients with height ≥ 165 cm was too small (Table 5). For higher parturient (women in Europe and America), the height based dosing algorithm of bupivacaine need to be further studied. Second, we used a small dose of bupivacaine in Test group, the sensory block level is lower (Table 4) and may recover to < T8 more quickly (20). In some parturients, analgesic via a vein or local anesthetic via the epidural space needs to be timely supplied. In addition, opiates (morphine, fentanyl, and sufentanil) may be applied into subarachnoid space to improve the quality of analgesia (43, 44). We did not inject the opiates and bupivacaine together, because we did not observe an obvious improvement in quality of analgesia after adding the opiates in our practice. This is consistent to Siddiqui et al.'s study (30).

## Conclusions

The dose of bupivacaine depended on height; 0.5% bupivacaine (1.15–1.7 ml, isobaric) varying with the height (0.05 ml/2–3 cm) is a suitable algorithm; the height based dosing algorithm of bupivacaine provided sufficient anesthesia with a low incidence of hypotension in the case of no prophylactic fluid preloading and vasopressors.

## Data Availability Statement

The original contributions presented in the study are included in the article/supplementary material, further inquiries can be directed to the corresponding author/s.

## Ethics Statement

The studies involving human participants were reviewed and approved by Ethics Committee of Shenzhen People's Hospital of Jinan University. The patients/participants provided their written informed consent to participate in this study.

## Author Contributions

BH and ZH conceived, designed, and revised the experiments. BH, QH, CH, and ZZ performed the experiments. QH, GW, and YL analyzed the data. ZH contributed reagents, materials, and analysis tools. BH wrote the paper. All authors contributed to the article and approved the submitted version.

## Funding

This work was supported by grants from the Doctoral Innovation Program of Health and Family Planning Commission of Shenzhen Municipality of China (SZBC2017016) to BH.

## Conflict of Interest

The authors declare that the research was conducted in the absence of any commercial or financial relationships that could be construed as a potential conflict of interest.

## Publisher's Note

All claims expressed in this article are solely those of the authors and do not necessarily represent those of their affiliated organizations, or those of the publisher, the editors and the reviewers. Any product that may be evaluated in this article, or claim that may be made by its manufacturer, is not guaranteed or endorsed by the publisher.
